# Preliminary evaluation of the publicly available Laboratory for Breast Radiodensity Assessment (LIBRA) software tool: comparison of fully automated area and volumetric density measures in a case–control study with digital mammography

**DOI:** 10.1186/s13058-015-0626-8

**Published:** 2015-08-25

**Authors:** Brad M. Keller, Jinbo Chen, Dania Daye, Emily F. Conant, Despina Kontos

**Affiliations:** 1 Department of Radiology, Perelman School of Medicine, University of Pennsylvania, 1 Silverstein Building, 3400 Spruce Street, Philadelphia, PA 19104 USA; 2 Department of Biostatistics and Epidemiology, Perelman School of Medicine, University of Pennsylvania, 203 Blockley Hall, 423 Guardian Drive, Philadelphia, PA USA

## Abstract

**Introduction:**

Breast density, commonly quantified as the percentage of mammographically dense tissue area, is a strong breast cancer risk factor. We investigated associations between breast cancer and fully automated measures of breast density made by a new publicly available software tool, the Laboratory for Individualized Breast Radiodensity Assessment (LIBRA).

**Methods:**

Digital mammograms from 106 invasive breast cancer cases and 318 age-matched controls were retrospectively analyzed. Density estimates acquired by LIBRA were compared with commercially available software and standard Breast Imaging-Reporting and Data System (BI-RADS) density estimates. Associations between the different density measures and breast cancer were evaluated by using logistic regression after adjustment for Gail risk factors and body mass index (BMI). Area under the curve (AUC) of the receiver operating characteristic (ROC) was used to assess discriminatory capacity, and odds ratios (ORs) for each density measure are provided.

**Results:**

All automated density measures had a significant association with breast cancer (OR = 1.47–2.23, AUC = 0.59–0.71, *P* < 0.01) which was strengthened after adjustment for Gail risk factors and BMI (OR = 1.96–2.64, AUC = 0.82–0.85, *P* < 0.001). In multivariable analysis, absolute dense area (OR = 1.84, *P* < 0.001) and absolute dense volume (OR = 1.67, *P* = 0.003) were jointly associated with breast cancer (AUC = 0.77, *P* < 0.01), having a larger discriminatory capacity than models considering the Gail risk factors alone (AUC = 0.64, *P* < 0.001) or the Gail risk factors plus standard area percent density (AUC = 0.68, *P* = 0.01). After BMI was further adjusted for, absolute dense area retained significance (OR = 2.18, *P* < 0.001) and volume percent density approached significance (OR = 1.47, *P* = 0.06). This combined area-volume density model also had a significantly (*P* < 0.001) improved discriminatory capacity (AUC = 0.86) relative to a model considering the Gail risk factors plus BMI (AUC = 0.80).

**Conclusions:**

Our study suggests that new automated density measures may ultimately augment the current standard breast cancer risk factors. In addition, the ability to fully automate density estimation with digital mammography, particularly through the use of publically available breast density estimation software, could accelerate the translation of density reporting in routine breast cancer screening and surveillance protocols and facilitate broader research into the use of breast density as a risk factor for breast cancer.

**Electronic supplementary material:**

The online version of this article (doi:10.1186/s13058-015-0626-8) contains supplementary material, which is available to authorized users.

## Introduction

It is increasingly recommended that breast cancer screening recommendations be personalized on the basis of a woman’s risk for breast cancer [1, 2]. This risk is known to be influenced by a number of factors, such as reproductive history [3], family history [4], body mass index (BMI) [5], and genetic traits [6], which form the basis for most current breast cancer risk assessment models [7]. The National Cancer Institute’s risk assessment tool, the Gail model [8], is one of the most commonly used risk assessment models and incorporates primarily reproductive and familial risk factors [7, 8]. The Gail model has several advantages, including being validated in large populations and for different racial groups [8–10], based on relatively simple-to-assess risk factors. However, though shown to be well calibrated at the population level, it has only moderate discriminatory accuracy at the individual level [11], limiting its use for personalized clinical decision making.

An opportunity for improving the discriminatory accuracy of risk assessment models is the incorporation of breast density [12]. Typically estimated via visual assessment either qualitatively by using the American College of Radiology Breast Imaging-Reporting and Data System (BI-RADS) density categories [13] or quantitatively as percent density (PD %) [14], mammographic density has been consistently shown to be an independent risk factor for breast cancer [14–22], potentially the strongest after age [14]. This has led to the development of several fully automated breast density algorithms such as the automated ImageJ method [23] and the standardized measures of area density proposed by Heine et al. [24]. More recently, measures of the volumetric amount of dense tissue have been proposed as more accurate representations of the underlying fibroglandular tissue content and, as such, potentially better predictors of risk [21, 25–29].

To date, studies evaluating the use of volumetric versus area density measures in breast cancer risk assessment have focused primarily on determining which individual measure is a better predictor of risk and have reported mixed results [19–21, 25, 26]. Here, we investigate both individual as well as joint associations between quantitative estimates of volumetric and area density and invasive breast cancer. As a first step, we examine these associations in a case–control setting after adjusting for standard risk factors and BMI. By establishing these associations and evaluating their magnitude, our study provides instrumental evidence toward identifying new promising density measures to be considered in breast cancer risk assessment models [30] for improving their discriminatory accuracy. In addition, our study is the first to evaluate associations between breast cancer and density measures as estimated by a new publically available breast density estimation software tool [31]. Our long-term hypothesis is that volumetric and area-based density measures, when considered jointly with standard risk factors and BMI, can independently contribute to and improve breast cancer risk assessment, compared with percent density measures or standard risk factors alone.

## Methods

### Study population

In this Health Insurance Portability and Accountability Act-compliant study approved by the institutional review board at the University of Pennsylvania (protocol #814186), we retrospectively identified women whose unilateral, invasive breast cancer was diagnosed at age 40 years or older from a previously completed multimodality breast imaging trial in our institution (2002–2006; National Institutes of Health P01 CA85484). The trial recruited a total of 901 women, each of whom met at least one of the following inclusion criteria: they were presenting for staging with newly diagnosed breast cancer, or they had a mammographically detected suspicious finding (BI-RADS of at least 4) after screening or diagnostic evaluation (or both) and were directed to biopsy, or they had an otherwise-suspicious palpable mass directed to biopsy, or they were evaluated to be at a high risk for developing breast cancer as determined by an estimated high lifetime risk of more than 25 % (using either the Gail or Claus risk models), or they had a recently diagnosed contralateral breast cancer. All women received an array of breast imaging modalities, including digital mammography. Informed written consent was obtained prior to study participation. From these women, 317 were diagnosed with primary breast cancer, of which 231 were invasive. Of the 231 women diagnosed with invasive breast cancer, 146 had raw (i.e., “For Processing”) digital mammograms available for analysis (Senographe 2000D and DS; GE Healthcare, Little Chalfont, UK). From this subset of 146 women, 10 were excluded for having bilateral breast cancer and thus not having a cancer-unaffected mammogram available for our analysis, and eight were excluded for having mammograms with artifacts or insufficient image quality (i.e., blur, motion effects, etc.), preventing valid breast density estimates from being obtained accurately with our automated software. An additional 22 women were excluded for being under the age of 40 and therefore not representative of a standard screening population. Thus, a total of 106 women with invasive cancer and no personal history of breast cancer were included in our analysis. No statistically significant differences in risk factor distributions between the included and total excluded women in this study were observed (*P* > 0.05).

Controls were randomly selected asymptomatic women who had breast cancer screening with digital mammography in our institution over the closest possible overlapping time period (2005–2006) where digital mammography was available to the general screening population and had raw digital mammograms available. Controls were matched to cases at a 1:3 ratio at 5-year age intervals. This resulted in 318 controls available for analysis, yielding a total cohort size of 424 women (average age of 55.1 ± 8.9 years). For density estimation, we used the mediolateral oblique (MLO) view mammogram of the contralateral, unaffected breast of cases as a surrogate of inherent breast tissue properties; MLO mammograms of controls were subsequently side-matched to cases. For this retrospective analysis, the requirement of informed consent was waived under institutional review board approval.

### Demographic risk factors

To account for possible confounding of the breast density measures by other known risk factors, we also considered self-reported information regarding standard demographic and reproductive risk factors for breast cancer [8]. For cancer cases, this information was available from the previously completed imaging trial. For controls, this information was obtained from screening questionnaires collected at the time at which the mammograms used in our study were acquired.

As the Gail model [8] is the current standard for assessing a woman’s risk for breast cancer, we abstracted the available demographic and reproductive information to match, as closely as possible, the risk factor coding used by the Gail model [8] (Table 1). Age was treated as a categorical variable based on 5-year age intervals, as in the matching process used during the study design. Age at menarche, number of previous benign biopsies, and first-degree family history (i.e., the number of first-degree female relatives with breast cancer) were treated as ordinal variables with the lowest value chosen as the reference group. For age at first live birth (i.e., parity), the Gail model considers whether it occurred prior to age 20, between age 20 and 24, 25 to 29, or after age 29 (i.e., 30 years or older), while nulliparous women are included in the 25- to 29-year age group as they have similar ORs for breast cancer [8]. To approximate the Gail model encoding given that we had only the following three descriptive categories available for age at first live birth in our screening questionnaires, we encoded parity as a nominal variable, comparing women in the under 30-year group and 30-year-or-older group versus nulliparous women as the reference group. We also adjusted for race as a nominal variable by using Caucasian women as the reference group to reflect the Gail model, which has been validated in Caucasian [8], African-American [9], and Asian [10] populations.

**Table 1:** Baseline demographics and breast density estimates by case–control status

	Controls	Cases	*P* value
Number of women	318	106	
Age at menarche			0.02*
Reference group: <12 years (0)	34 (13 %)	26 (25 %)	
12–14 years (1)	197 (75 %)	71 (67 %)	
15 years or older (2)	32 (12 %)	9 (8 %)	
Missing	55	0	
Number of previous benign biopsies			0.36
Reference group: 0 (0)	235 (74 %)	74 (70 %)	
1 (1)	53 (17 %)	24 (23 %)	
2+ (2)	30 (9 %)	8 (8 %)	
Missing	0	0	
Number of first-degree relatives with breast cancer			0.89
Reference group: 0 (0)	242 (77 %)	83 (78 %)	
1 (1)	65 (21 %)	22 (21 %)	
2+ (2)	5 (2 %)	1 (1 %)	
Missing	6	0	
Age at first live birth			0.08
Reference group: Nulliparous (0)	252 (27 %)	17 (16 %)	
Before age 30 (1)	167 (53 %)	63 (60 %)	
Age 30 or older (2)	65 (20 %)	26 (25 %)	
Missing	1	0	
Race			0.22
Reference group: Caucasian (0)	162 (64 %)	79 (75 %)	
African American (1)	72 (29 %)	21 (20 %)	
Asian (2)	10 (4 %)	2 (2 %)	
Other (3)	8 (3 %)	3 (3 %)	
Missing	66	1	
Body mass index			<0.001*
Reference group: Normal weight (0)	161 (59 %)	20 (24 %)	
Overweight (1)	75 (28 %)	24 (28 %)	
Obese (2)	36 (13 %)	40 (48 %)	
Missing	46	22	
BI-RADS breast density			0.65
Reference group: Predominantly fatty (0)	21 (7 %)	4 (4 %)	
Scattered fibroglandular densities (1)	134 (42 %)	50 (47 %)	
Heterogeneously dense (2)	160 (50 %)	51 (48 %)	
Extremely dense (3)	3 (1 %)	1 (1 %)	
LIBRA: absolute dense area, mean ± SD	31.3 cm^2^ ± 17.8	48.5 cm^2^ ± 29.4	<0.001*
LIBRA: area percent density, mean ± SD	27.1 % ± 14.7	31.9 % ± 15.5	<0.001*
Quantra: absolute dense volume, mean ± SD	73.1 cm^3^ ± 50.0	121.7 cm^3^ ± 98.9	<0.001*
Quantra: volume percent density, mean ± SD	11.6 % ± 6.4	13.6 % ± 6.9	0.002*

Pearson chi-squared test is used to test differences in demographic variables between cases and controls with known values. Two-sample *t* test is used to test for differences between log-transformed breast density estimates

The numeric coding for the categorical variables used as inputs in the regression models are provided in parentheses, with the ‘0’ groups representing the reference group for each categorical variable

*BI-RADS* Breast Imaging-Reporting and Data System, *LIBRA* Laboratory for Individualized Breast Radiodensity Assessment, *SD* standard deviation

*denotes statistical significance at the α = 0.05 level

In addition, BMI was abstracted from archived clinical records. Based on available height and weight information, women were classified as normal weight (BMI < 25 kg/m^2^), overweight (25 kg/m^2^ ≤ BMI < 30 kg/m^2^), or obese (30 kg/m^2^ ≤ BMI). In this study, BMI was treated as an ordinal variable.

### Breast density measures

**Fig. 1:**
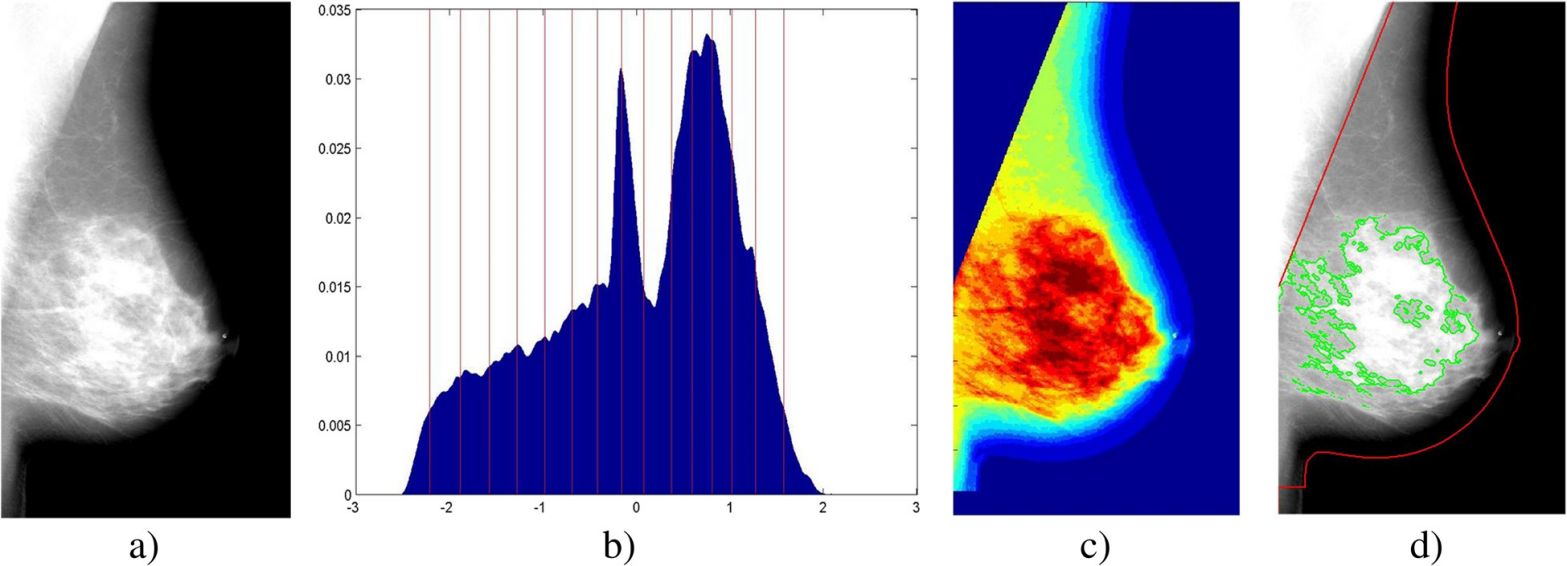
Example of density segmentation using the LIBRA software tool. **a** Left mediolateral oblique “For Processing” raw mammogram of a 57-year-old woman with a negative screening exam. **b** Breast image intensity histogram with fuzzy c-means clustering centroids (vertical lines). **c** Intensity-clustered breast image. **d** The final breast and dense tissue segmentation. *LIBRA* Laboratory for Individualized Breast Radiodensity Assessment

Breast density was measured by using fully automated software. Absolute dense area and area percent density (PD %) were estimated by using a publically available software tool [31], the Laboratory for Individualized Breast Radiodensity Assessment (LIBRA), based on our previously proposed adaptive multi-cluster fuzzy c-means segmentation algorithm [32]. The LIBRA algorithm has been previously validated against the current standard semi-automated Cumulus method [33], showing similar agreement for both raw (i.e., “For Processing”) and vendor post-processed (i.e., “For Presentation”) digital mammograms (Fig. 1) [32], for the same vendor used in this study. Briefly, the algorithm first applies an edge-detection algorithm to delineate the boundary of the breast and the pectoral muscle. An adaptive multi-class fuzzy c-means algorithm is applied to identify and partition the image gray levels (Fig. 1b) within the mammographic breast tissue area, *B*_*A*_, into regions (i.e., clusters) of similar x-ray attenuation (Fig. 1c). These clusters are then aggregated by a support-vector machine classifier to a final absolute dense area, *D*_*A*_, segmentation (Fig. 1d). The ratio of the absolute dense area to the total breast area is used to obtain a measure of breast percent density (PD %):

1 $$ \mathrm{P}\mathrm{D}\%=\frac{D_A}{B_A} $$

Absolute dense tissue volume and volume percent density (VD %) were automatically assessed by using FDA-cleared software (Quantra™ version 2.0; Hologic Inc., Bedford, MA, USA). The algorithm is based on the widely validated method of Highnam et al. [34] adapted for digital mammography [35]. Briefly, this method quantifies the thickness of dense (i.e., fibroglandular) tissue within each image pixel based on physical parameters of the breast and the imaging system as well as on imaging physics of individual exposures, such as attenuation coefficients for breast tissue, x-ray spectra for the target material, x-ray energy (i.e., peak kilovoltage), exposure, and organ dose (i.e., decigray). Aggregation of the per-pixel volumes for the entire breast allows estimation of the total breast volume, *B*_*V*_, and dense tissue volume, *D*_*V*_. The ratio of absolute dense tissue volume to absolute breast volume provides a measure of VD % as:

2 $$ \mathrm{V}\mathrm{D}\%=\frac{D_V}{B_V} $$

Lastly, for comparison with the automated density measures, we also obtained standard four-category BI-RADS density estimates via retrospective review of archived clinical reports, in which the density assessment was made at the time of routine clinical evaluation by the interpreting breast radiologist for that individual mammography study.

### Statistical analysis

The continuous breast density measures were first log-transformed to account for their skewed distributions. Differences in standard risk factors, BMI, and breast density distributions between cases and controls were assessed by using chi-squared tests for categorical variables and two-sided *t* tests for continuous variables at the standard α = 0.05 significance level. The associations between PD %, VD %, absolute dense area, and absolute dense volume were assessed by using linear regression and Spearman correlation, as were the associations between the four density measures and BMI. Univariate (i.e., unadjusted) and multivariable (i.e., fully adjusted) logistic regression was performed to assess the association between the different log-transformed breast density measures and breast cancer before and after adjusting for the standard risk factors and BMI. Odds ratios (ORs), 95 % confidence intervals (CIs), and significance were estimated for all risk factors. For categorical variables, we used a test-for-trend analysis, except for race and parity which were treated as nominal variables. ORs for continuous density measures are reported per standard deviation increase based on the distribution of the breast density metrics in the controls. Missing risk factors (Table 1) were accounted for by standard multiple imputation by using bootstrapping [36] stratified by cancer status, in which 25 imputations were used for this analysis, which is greater than the suggested minimum number of 20.

The area under the curve (AUC) of the receiver operating characteristic (ROC) was used to evaluate the discriminatory capacity of each unadjusted and adjusted breast density model [30]. The adjusted density models are then compared with baseline models fit only on standard risk factors before and after the inclusion of BMI by using DeLong’s test [37]. In addition, we compared the performance of the area and volume method of each type (i.e., absolute and relative percent) by using DeLong’s test to determine whether choice of the density measure influences discriminatory capacity. Finally, as the different breast density measures were expected to be moderately correlated [21], we applied backward stepwise feature selection with standard variable entry and removal criterion (*P*_enter_ < 0.05; *P*_removal_ > 0.1) commonly used in the literature [38] both to the risk factor-adjusted and fully adjusted models, to identify those measures of breast density with statistically independent associations to breast cancer while simultaneously mitigating the risk of over-parameterizing the individual models [39]. These feature-selected, adjusted main-effects models were then compared with models fitted only on standard risk factors and a model including standard risk factors and area percent density (i.e., PD %), before and after the inclusion of BMI. The performance of these models was also compared by using DeLong’s test. All analyses were performed in Stata 13.1 (StataCorp LP, College Station, TX, USA).

## Results

### Comparison of risk factors between cases and controls

Women in case and control groups were similar in terms of number of prior benign biopsies (*P* = 0.36), first-degree family history (*P* = 0.89), race (*P* = 0.22), and age at first live birth (*P* = 0.08) while being significantly different in terms of their BMI (*P* < 0.001) and age at menarche (*P* = 0.02). In terms of mammographic density estimated via automated software, higher absolute density estimates were significantly associated with cancer status (*P* < 0.001), as were the percent density estimates (*P* ≤ 0.002), regardless of whether area or volumetric density measures were considered, whereas BI-RADS density estimates were not significant (*P* = 0.65). Table 1 provides a summary of standard risk factors and breast density measures for our case–control groups.

### Relationship between area and volumetric breast density

**Fig. 2:**
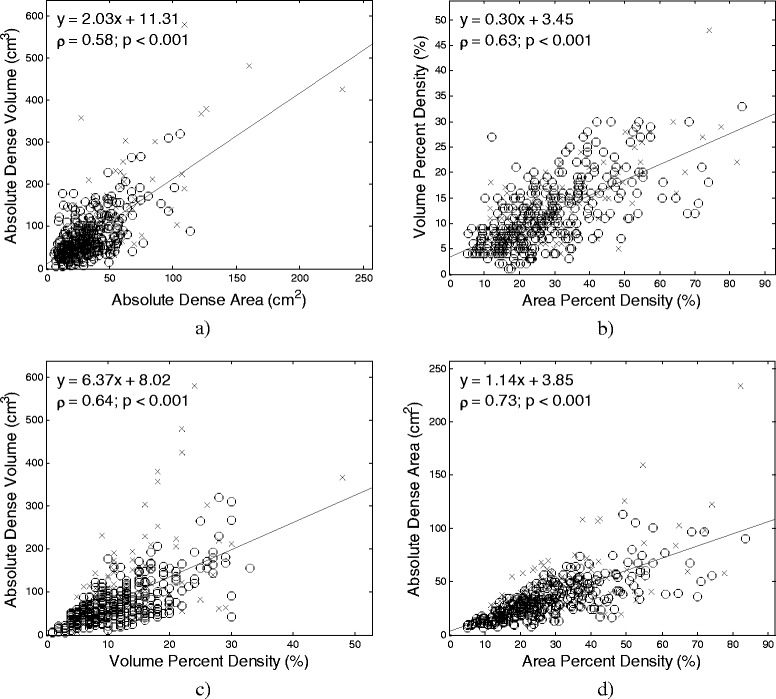
Relationship between different breast density measures. The association between the area-based and volumetric breast density is provided for both (**a**) absolute measures and (**b**) percent measures. The relationship between absolute and percent breast density measures are shown for (**c**) volumetric and (**d**) area density. Cancer cases are demarcated by ‘x’, controls by ‘o’. Regression lines, equations, and Spearman correlations are provided for reference

Statistically significant (*P* < 0.001) correlations were observed between the different quantitative density estimates (Spearman correlation: ρ = 0.24–0.73). Area percent density and absolute dense area had the strongest correlation (ρ = 0.73, 95 % CI 0.69–0.78). Absolute dense volume and area percent density had the weakest correlation (ρ = 0.24, 95 % CI 0.15–0.33). Figure 2 shows correlation and linear regression plots for the different area and volumetric breast density measures.

### Relationship between breast density measures and body mass index

**Fig. 3:**
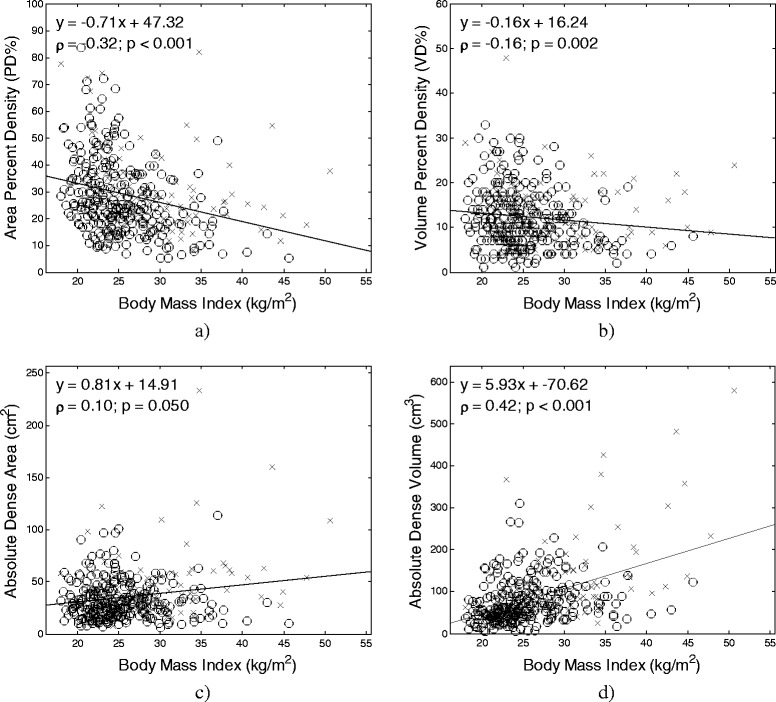
Relationship between BMI and breast density measures. The association between area-based and volumetric breast density versus BMI is provided for (**a**) area percent density (PD %), (**b**) volume percent density, (**c**) absolute dense area and (**d**) absolute dense volume. Cancer cases are demarcated by ‘x’; controls by ‘o’. Regression lines, equations, and Spearman correlations are also provided for reference. *BMI* body mass index

Statistically significant (*P* < 0.05) correlations were observed between the different quantitative density estimates and BMI (Spearman correlation: ρ = −0.31–0.42). Absolute dense volume and BMI had the strongest correlation (ρ = 0.42, 95 % CI 0.38–0.53), whereas absolute dense area and BMI had the weakest correlation (ρ = 0.10, 95 % CI 0.00–0.21). Figure 3 shows correlation and linear regression plots for the different area and volumetric breast density measures versus BMI.

### Associations between area and volumetric breast density and breast cancer

Univariate analysis of the standard risk factors (Table 2) shows that BMI (*P* < 0.001) and age at menarche (*P* = 0.01) were significantly associated with breast cancer for our study population, with later age at menarche being a protective factor (OR = 0.57, 95 % CI 0.37–0.88) and increasing BMI a risk factor (OR = 2.97, 95 % CI 2.17–4.05). Parity after age 30 years was also observed to be a risk factor (OR = 2.00, 95 % CI 1.00–3.99, *P* = 0.05), as was parity prior to age 30 (OR = 1.88, 95 % CI 1.04–3.42, *P* = 0.04). Before adjustment for the standard Gail risk factors and BMI, all four quantitative breast density measures were statistically significantly associated with breast cancer at the standard level (*P* ≤ 0.002; Table 3). Absolute measures of breast density had a higher discriminatory capacity in distinguishing cancer status (AUC = 0.68–0.71) than percent density measures (AUC = 0.59–0.60; *P* < 0.05) or the Gail risk factors (Table 2: AUC = 0.51–0.60; *P* < 0.05) but not higher than BMI (AUC = 0.76). In addition, the discriminatory capacity of absolute dense area and absolute dense volume were not found to be significantly different (*P* = 0.38), nor were area percent density and volume percent density (*P* = 0.63).

**Table 2:** Unadjusted odds ratios, *P* values and discriminatory capacity (AUC) for the standard risk factors considered for our study population

Risk factor	Odds ratio	*P* value	AUC
Age at menarche (ordinal)			0.54 (0.49–0.60)
Reference group: under age 12 years	1	–	
	(N/A)		
Between ages 12 and 14 years	0.57	0.01	
	(0.37–0.88)		
Age 15 years and older	0.32	0.01	
	(0.13–0.78)		
Number of benign biopsies (ordinal)			0.52 (0.47–0.57)
Reference group: 0 biopsies	1	–	
	(N/A)		
1 biopsy	1.05	0.76	
	(0.75–1.48)		
2 or more biopsies	1.11	0.76	
	(0.56–2.19)		
First-degree family history (ordinal)			0.51 (0.46–0.56)
Reference group: 0 relatives	1	–	
	(N/A)		
1 relative	0.93	0.77	
	(0.57–1.51)		
2 or more relatives	0.86	0.77	
	(0.33–2.29)		
Age at first live birth (nominal)			0.56 (0.50–0.61)
Reference group: nulliparous women	1	–	
	(N/A)		
Prior to age 30 years	1.88	0.04	
	(1.04–3.42)		
At age 30 years or older	2.00	0.05	
	(1.00–3.99)		
Race (nominal)			0.60 (0.54–0.65)
Reference group: Caucasian	1	–	
	(N/A)		
African-American	0.60	0.07	
	(0.34–1.03)		
Asian	0.40	0.24	
	(0.08–1.84)		
Other	0.78	0.72	
	(0.20–3.04)		
Body mass index (ordinal)			0.76 (0.71–0.81)
Reference group: normal	1	–	
	(N/A)		
Overweight	2.97	<0.001	
	(2.17–4.05)		
Obese	8.81	<0.001	
	(4.72–16.4)		

Parentheses indicate whether the variable was treated as binary, ordinal, or nominal. Ninety-five percent confidence intervals are provided in parenthesis

*AUC* area under the curve, *N/A* not applicable

**Table 3:** Unadjusted odds ratios per standard deviation increase in the log-transformed LIBRA and Quantra density measures (per-category in the ordinal BI-RADS density measure), *P* values, and discriminatory capacity (AUC) of the continuous and categorical BI-RADS breast density estimates

Density measure	Odds ratio	*P* value	AUC	Odds ratios at specific percentiles/Categories			
				10th	50th	75th	90th
LIBRA: absolute dense area, cm^2^	2.23	<0.001	0.71^a^	1	3.33	5.81	8.38
(Continuous)	(1.72–2.88)		(0.65–0.76)	(N/A - reference)	(2.25–4.88)	(3.28–10.17)	(4.21–16.52)
LIBRA: area percent density, PD %	1.50	<0.001	0.60^a^	1	1.66	2.17	2.71
(Continuous)	(1.18–1.91)		(0.54–0.66)	(N/A - reference)	(1.23–2.24)	(1.37–3.44)	(1.5–4.91)
Quantra: absolute dense volume, cm^3^	2.04	<0.001	0.68^a^	1	2.41	4.05	5.87
(Continuous)	(1.58–2.63)		(0.62–0.74)	(N/A - reference)	(1.76–3.3)	(2.45–6.66)	(3.11–11.03)
Quantra: volume percent density, VD %	1.47	0.002	0.59^a^	1	1.65	2.09	2.48
(Continuous)	(1.15–1.89)		(0.53–0.65)	(N/A - reference)	(1.2–2.28)	(1.31–3.37)	(1.39–4.48)
Radiologist’s BI-RADS density (ordinal categories)	1.02 (0.71–1.45)	0.93	0.50 (0.44–0.55)	Predominantly fatty	Scattered fibroglandular densities	Heterogeneously dense	Extremely dense
				1	1.02	1.03	1.05
				(N/A - reference)	(0.71–1.45)	(0.51–2.10)	(0.36–3.04)

Odds ratios computed at specific corresponding percentiles of the distribution of the LIBRA and Quantra density estimates relative to the 10th percentile and at the specific categories for BI-RADS density are also provided as a reference. Ninety-five percent confidence intervals of the odds ratios are also provided in parenthesis below the point estimate

*LIBRA* Laboratory for Individualized Breast Radiodensity Assessment, *BI-RADS* Breast Imaging-Reporting and Data System, *AUC* area under the curve, *N/A* not applicable

^a^indicates a statistically significant AUC

1

After adjustment for the Gail risk factors (Table 4), the associations between all four measures and cancer status remained significant and were strengthened, and the discriminatory capacity of the models including absolute measures of density (AUC = 0.74–0.75) was significantly improved (*P* < 0.003) relative to the model including only Gail risk factors. As with the unadjusted models, no difference was observed in the discriminatory capacity between the absolute (*P* = 0.57) or percent (*P* = 0.78) area and volume density measures. Finally, when both the standard Gail risk factors and BMI were adjusted for (Table 5), all four density measures retained statistical significance (*P* < 0.001), and most had strengthened associations with breast cancer relative to their unadjusted associations as evidenced by the lower *P* values, or higher ORs, or both. The discriminatory capacities of the absolute dense area (AUC = 0.85), percent dense area (AUC = 0.85), and percent dense volume (AUC = 0.83) metrics were also significantly (*P* ≤ 0.005) higher than the baseline standard Gail risk factors and BMI model (AUC = 0.80). Furthermore, after adjustment for both Gail risk factor information and BMI, absolute dense area was found to have a significantly (*P* = 0.009) larger discriminatory capacity (AUC = 0.85) relative to absolute dense volume (AUC = 0.82), whereas the difference between the area and volume percent density measures only approached significance (*P* = 0.08). In contrast, BI-RADS density was not found to be significantly associated with breast cancer in our study population either before or after adjustment for other risk factors.

**Table 4:** Odds ratios per standard deviation increase in the log-transformed LIBRA and Quantra density measures (per-category in the ordinal BI-RADS density measure) adjusted for the Gail risk factors (i.e., age at menarche, parity, family history, age, race and number of prior biopsies), *P* values, and discriminatory capacity (AUC) of the continuous and categorical BI-RADS breast density estimates

Density measure	Odds ratio	*P* value	AUC	Odds ratios at specific percentiles/Categories			
				10th	50th	75th	90th
LIBRA: absolute dense area, cm^2^	2.45	<0.001	0.75^a^	1	3.83	7.14	10.76
(Continuous)	(1.86–3.24)		(0.70–0.81)	(N/A - reference)	(2.54–5.82)	(3.9–13.17)	(5.18–22.58)
LIBRA: area percent density, PD %	1.64	<0.001	0.68	1	1.85	2.57	3.37
(Continuous)	(1.25–2.14)		(0.62–0.74)	(N/A - reference)	(1.32–2.58)	(1.53–4.28)	(1.73–6.49)
Quantra: absolute dense volume, cm^3^	2.40	<0.001	0.74^a^	1	2.95	5.57	8.79
(Continuous)	(1.80–3.21)		(0.68–0.79)	(N/A - reference)	(2.07–4.22)	(3.17–9.85)	(4.3–18.1)
Quantra: volume percent density, VD %	1.68	<0.001	0.68	1	1.96	2.69	3.39
(Continuous)	(1.09–1.72)		(0.62–0.74)	(N/A - reference)	(1.12–2.02)	(1.18–2.82)	(1.22–3.59)
Radiologist’s BI-RADS density (ordinal categories)	1.07 (0.74–1.56)	0.71	0.64 (0.58–0.71)	Predominantly fatty	Scattered fibroglandular densities	Heterogeneously dense	Extremely dense
				1	1.07	1.15	1.23
				(N/A - reference)	(0.74–1.56)	(0.55–2.43)	(0.41–3.80)

Odds ratios computed at specific corresponding percentiles of the distribution of the LIBRA and Quantra density estimates relative to the 10th percentile and at the specific categories for BI-RADS density are also provided as a reference. Ninety-five percent confidence intervals of the odds ratios are also provided in parenthesis below the point estimate

*LIBRA* Laboratory for Individualized Breast Radiodensity Assessment, *BI-RADS* Breast Imaging-Reporting and Data System, *AUC* area under the curve, *N/A* not applicable

^a^indicates that the AUC of a model including the specific density measure has a statistically significant increase in discriminatory capacity over a baseline model including only standard risk factors (AUC = 0.64)

**Table 5:** Odds ratios per standard deviation increase in the log-transformed LIBRA and Quantra density measures (per-category in the ordinal BI-RADS density measure) adjusted for both BMI and the Gail risk factors (i.e., age at menarche, parity, family history, age, race and number of prior biopsies), *P* values, and discriminatory capacity (AUC) of the continuous and categorical BI-RADS breast density estimates

Density measure	Odds ratio	*P* value	AUC	Odds ratios at specific percentiles/Categories			
				10th	50th	75th	90th
LIBRA: absolute dense area, cm^2^	2.57	<0.001	0.85^a^	1	4.12	7.92	12.22
(Continuous)	(1.86–3.56)		(0.81–0.90)	(N/A - reference)	(2.54–6.71)	(3.9–16.19)	(5.18–28.98)
LIBRA: area percent density, PD %	2.64	<0.001	0.85^a^	1	3.35	6.39	10.88
(Continuous)	(1.79–3.89)		(0.81–0.90)	(N/A - reference)	(2.07–5.44)	(3.04–13.41)	(4.18–28.21)
Quantra: absolute dense volume, cm^3^	1.96	<0.001	0.82	1	2.3	3.74	5.32
(Continuous)	(1.44–2.67)		(0.77–0.86)	(N/A - reference)	(1.57–3.36)	(2.04–6.86)	(2.47–11.46)
Quantra: volume percent density, VD %	2.24	<0.001	0.83^a^	1	2.84	4.66	6.68
(Continuous)	(1.56–3.21)		(0.78–0.87)	(N/A - reference)	(1.78–4.52)	(2.34–9.26)	(2.85–15.58)
Radiologist’s BI-RADS density (ordinal categories)	1.06 (0.69–1.63)	0.78	0.80 (0.75–0.85)	Predominantly fatty	Scattered fibroglandular densities	Heterogeneously dense	Extremely dense
				1	1.06	1.12	1.19
				(N/A - reference)	(0.69–1.63)	(0.48–2.66)	(0.33–4.33)

Odds ratios computed at specific corresponding percentiles of the distribution of the LIBRA and Quantra density estimates relative to the 10^th^ percentile and at the specific categories for BI-RADS density are also provided as a reference. Ninety-five percent confidence intervals of the odds ratios are also provided in parenthesis below the point estimate

*LIBRA* Laboratory for Individualized Breast Radiodensity Assessment, *BI-RADS* Breast Imaging-Reporting and Data System, *BMI* body mass index, *AUC* area under the curve, *N/A* not applicable

^a^indicates that the AUC of a model including the specific density measure has a statistically significant increase in discriminatory capacity over a baseline model including only standard risk factors and BMI (AUC = 0.80)

In multivariable analysis (Table 6), a logistic regression model based only on standard Gail risk factors had an AUC of 0.64 (95 % CI 0.58–0.71). A model combining these risk factors with the standard area percent density (i.e., PD %) had an improved discriminatory capacity (AUC = 0.68, 95 % CI 0.62–0.74), which was statistically different from the model including only the standard risk factors (*P* = 0.03). A combined, feature-selected model considering all four different breast density measures in combination with the Gail risk factors had an AUC of 0.77 (95 % CI 0.71–0.82), which was significantly higher than both the model including only the Gail risk factors (*P* < 0.001) and the model including the Gail risk factors plus area percent density (*P* < 0.001). In this combined model, absolute dense volume and absolute dense tissue area were jointly significant predictors of breast cancer (*P* ≤ 0.003), while area percent density, volume percent density, and BI-RADS density estimates were not retained (*P* > 0.1).

**Table 6:** Logistic regression models and discriminatory capacity before and after inclusion of the breast density estimates to the Gail risk factors

Risk factor	Standard risk factors only			Gail risk factors plus area percent density			Gail risk factors plus area and volumetric density with feature selection		
	Odds ratio	*P* value	AUC	Odds ratio	*P* value	AUC	Odds ratio	*P* value	AUC
Demographics			0.64 (0.58–0.71)			0.68^a^ (0.62–0.74)			0.77^a^ (0.71–0.82)
Age	0.96	0. 59		1.02	0.73		1.06	0.41	
	(0.84–1.10)			(0.90–1.17)			(0.92–1.23)		
Age at menarche	0.56	0.01		0.54	0.008		0.64	0.06	
	(0.36–0.89)			(0.34–0.85)			(0.40–1.02)		
Number of benign biopsies	1.08	0.67		1.02	0.94		0.97	0.90	
	(0.75–1.55)			(0.70–1.47)			(0.65–1.45)		
First-degree family history	0.91	0.72		0.83	0.49		0.72	0.26	
	(0.54–1.52)			(0.49–1.40)			(0.42–1.26)		
Parity					–			–	
Nulliparous	1	–		1	–		1	–	
	(N/A)			(N/A)			(N/A)		
Prior to age 30 years	2.22	0.01		2.49	0.005		3.33	0.001	
	(1.18–4.16)			(1.31–4.73)			(1.67–6.64)		
Age 30 years or older	2.01	0.05		2.12	0.04		2.46	0.02	
	(0.99–4.08)			(1.03–4.35)			(0.–4.08)		
Race									
Caucasian	1	–		1	–		1	–	
	(N/A)			(N/A)			(N/A)		
African-American	0.54	0.04		0.58	0.08		0.39	0.005	
	(0.30–0.97)			(0.32–1.06)			(0.21–0.75)		
Asian	0.45	0.33		0.50	0.40		0.55	0.49	
	(0.09–2.19)			(0.10–2.52)			(0.10–2.98)		
Other	0.80	0.76		0.90	0.88		1.04	0.96	
	(0.20–3.27)			(0.21–3.83)			(0.23–4.74)		
Continuous density estimates									
LIBRA: absolute dense area	–	–		–	–		1.84	<0.001	
							(1.32–2.54)		
LIBRA: area percent density	–	–		1.64	<0.001		–	–	
				(1.25–2.14)					
Quantra: absolute dense volume	–	–		–	–		1.67	0.003	
							(1.19–2.34)		
Quantra: volume percent density	–	–		–	–		–	–	

Odds ratios, area under the curve (AUC) of the receiver operating characteristic and 95 % confidence intervals for models based on standard risk factors alone, standard risk factors plus area percent density, and a combined model with standard risk factors, and both area and volumetric density measures after feature selection are provided. Dashes indicate features not included in each model

*N/A* not applicable, *LIBRA* Laboratory for Individualized Breast Radiodensity Assessment

^a^indicates that the predictive capacity of a model including density is statistically significant than the baseline model including only Gail risk factors

Adjusting for BMI in addition to the Gail risk factors (Table 7) also led to increased discriminatory capacity in all models (AUC = 0.80–0.86). A logistic regression model based only on Gail and BMI risk factors had an AUC of 0.80 (95 % CI 0.75–0.85). The addition of the standard area percent density measure to the Gail-BMI risk factor model led to a statistically significant increase in performance (AUC = 0.85, *P* < 0.001). A combined, feature-selected model considering all four different breast density measures led to a statistically significant improvement in performance (AUC = 0.86) versus the baseline Gail-BMI model (*P* < 0.001) but not the Gail-BMI plus area percent density model (*P* = 0.64). In this combined model, absolute dense area was retained as a significant predictor after feature-selection (*P* < 0.001); volume percent density was also retained by the feature selection process as it approached significance (*P* = 0.06).

**Table 7:** Logistic regression models and discriminatory capacity before and after inclusion of the breast density estimates to a model containing both the Gail risk factors and body mass index

Risk factor	Standard risk factors and BMI			Standard risk factors and BMI plus area percent density			Standard risk factors and BMI plus area and volumetric density with feature selection		
	Odds ratio	*P* value	AUC	Odds ratio	*P* value	AUC	Odds ratio	*P* value	AUC
Demographics			0.80 (0.75–0.85)			0.85^a^ (0.81–0.90)			0.86^a^ (0.82–0.90)
Age	0.98	0.75		1.12	0.21		1.11	0.24	
	(0.83–1.14)			(0.94–1.33)			(0.93–1.32)		
Age at menarche	0.73	0.22		0.74	0.26		0.79	0.37	
	(0.44–1.20)			(0.44–1.24)			(0.47–1.32)		
Number of benign biopsies	1.19	0.39		1.10	0.67		1.07	0.75	
	(0.80–1.78)			(0.71–1.69)			(0.70–1.65)		
First-degree family history	0.89	0.68		0.78	0.42		0.74	0.33	
	(0.51–1.56)			(0.43–1.43)			(0.40–1.35)		
Parity									
Nulliparous	1	–		1	–		1	–	
	(N/A)			(N/A)			(N/A)		
Prior to age 30 years	2.08	0.04		2.46	0.02		2.88	0.006	
	(1.03–4.17)			(1.17–5.19)			(1.34–6.18)		
Age 30 years or older	2.00	0.09		2.03	0.09		2.27	0.06	
	(0.91–4.38)			(0.89–4.66)			(0.97–5.31)		
Race									
Caucasian	1	–		1	–		1	–	
	(N/A)			(N/A)			(N/A)		
African-American	0.30	0.001		0.29	0.004		0.24	0.001	
	(0.14–0.63)			(0.13–0.64)			(0.11–0.54)		
Asian	0.58	0.55		0.71	0.72		0.73	0.74	
	(0.10–3.37)			(0.11–4.72)			(0.11–4.66)		
Other	0.73	0.71		0.82	0.82		0.99	0.99	
	(0.15–3.69)			(0.14–4.66)			(0.18–5.46)		
BMI	3.43	<0.001		5.10	<0.001		3.93	<0.001	
	(2.33–5.04)			(3.07–8.45)			(2.47–6.26)		
Continuous density estimates									
LIBRA: absolute dense area	–	–		–	–		2.18	<0.001	
							(1.53–3.11)		
LIBRA: area percent density	–	–		2.64	<0.001		–	–	
				(1.79–3.89)					
Quantra: absolute dense volume	–	–		–	–		–	–	
Quantra: volume percent density	–	–		–	–		1.47	0.06	
							(0.99–2.19)		

Odds ratios, area under the curve (AUC) of the receiver operating characteristic (ROC), and 95 % confidence intervals for models based on standard risk factors alone, standard risk factors plus area percent density, and a combined model with standard risk factors, and both area and volumetric density measures after feature selection are provided. Dashes indicate features not included in each model

*BMI* body mass index, *N/A* not applicable, *LIBRA* Laboratory for Individualized Breast Radiodensity Assessment

^a^indicates that the predictive capacity of a model including density is statistically significant than the baseline model

Additional file 1: Table S1 and Additional file 2: Table S2 provide the full univariate logistic regression tables for absolute area, absolute volume, VD %, and BI-RADS breast density not already included in Tables 6 and 7 after adjustment for the standard Gail risk factors and BMI.

## Discussion

To date, compared with area-based measures of percent density which have been more broadly established as a risk factor, studies evaluating the use of volumetric density measures in breast cancer risk assessment have primarily focused on determining which individual measure is a better predictor of risk and have reported mixed results [19–21, 25, 26]. In this study, we investigated whether different quantitative measures of area and volumetric density are jointly and independently associated with breast cancer. Our results suggest that these novel, fully automated breast density measures may ultimately provide complementary information regarding breast cancer risk relative to both Gail risk factors and BMI. We also found that the strongest associations were obtained when absolute dense area, volume percent density, Gail risk factors, and BMI were considered in combination (AUC = 0.86, Table 7) and that this model yielded a statistically significant increase in discriminatory capacity over a model considering only the standard Gail risk factors and BMI (AUC = 0.80, *P* < 0.001). Overall, our study serves as a preliminary evaluation that will guide the design of larger prospective studies that will rigorously evaluate the predictive value of fully automated measures in risk assessment. In addition, our study is the first to evaluate the new, publically available LIBRA software [31] tool in terms of its association with breast cancer, providing preliminary evidence for its potential utility as an eventual marker of breast cancer risk.

Previous studies have investigated the potential value of breast density in breast cancer risk prediction, primarily using digitized film mammograms [22, 40–42]. Chen et al. found that incorporating percent density estimates into the Gail model leads to a moderate improvement in discriminatory capacity, with an AUC of 0.64 versus a baseline performance of 0.60 AUC without the inclusion of density [40]. Similarly, Tice et al. found that BI-RADS density can lead to a modest improvement in risk prediction (AUC = 0.66–0.68) in a pair of very large studies [41, 42]. However, although these studies have shown potential to improve risk prediction using density, discriminatory accuracy remains limited at the individual level [7]. There are several potential explanations. First, visual estimates of density are known to be highly variable among readers [43], affecting accuracy and standardization [14, 16]. In addition, most studies have not considered the volumetric amount of dense fibroglandular tissue but rather projection estimates of area percent density [27].

Studies comparing volumetric estimates of breast density to area-based measures have primarily focused on determining which single type of density measure is a better predictor of risk [20, 21, 25, 26] and have reported mixed results. Most of these studies suggest that increased volume of dense tissue is associated with an increased risk of breast cancer [20, 21, 25]; however, only in the study by Shepherd et al. [21] were the volumetric estimates of breast density found to be stronger predictors of risk than area-based percent density measures. This lack of concordance between studies may be due to several factors. First, most studies to date have analyzed populations with inherently different characteristics. In addition, most previous studies have quantified density by using digitized film mammograms, which are subject to additional sources of variation due to the digitization process and may require user interaction for obtaining the density estimates.

**Fig. 4:**
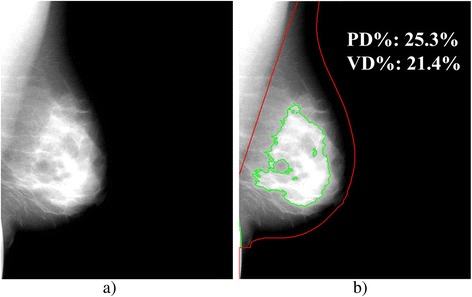
Mammogram of a breast consisting of a similar volumetric and area breast density. Example of (**a**) a mediolateral oblique view, “For Processing” (i.e., raw) mammogram and (**b**) the dense area tissue segmentation of a 56-year-old woman with a negative screening exam who has similar volumetric percent density (VD % = 21.4 %) and area breast percent density (PD % = 25.3 %) estimates

**Fig. 5:**
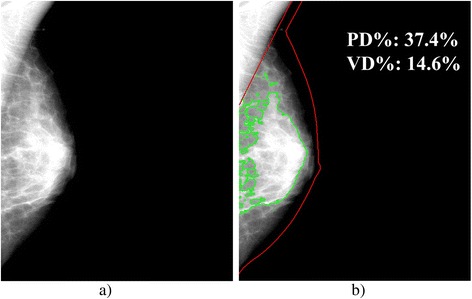
Mammogram of a breast consisting of a higher area breast density than volumetric density. Example of (**a**) a mediolateral oblique view, “For Processing” (i.e., raw) mammogram and (**b**) the dense area tissue segmentation of a 59-year-old woman with a negative screening exam who has different volumetric percent density (VD % = 14.6 %) and area breast percent density (PD % = 37.4 %) estimates

Although the etiological basis of breast density’s association to risk is not yet fully understood [44], an additional possibility is that both the total amount of glandular tissue in the breast captured by volumetric measures of breast density as well as the distribution of this tissue within the breast reflected by projection (i.e., area-based) measures of density may capture independent information regarding a woman’s risk for breast cancer. In this way, area and volume density measures could be considered components of the parenchymal pattern originally described visually by Wolfe in the 1970s [16, 45]. Wolfe’s patterns were designed in such a way so as to describe not only the amount of radio-opaque tissue in the breast (i.e., potentially best reflected by measures of volume density) but also its distribution throughout the breast by way of the ductal structures (i.e., potentially best reflected by measures of area density). This could, in turn, support our observation that both volumetric and area-based measures of density may be associated with breast cancer risk. For example, Fig. 4 shows a mammogram for which the volume percent density and area percent density are roughly equivalent in magnitude. In contrast, Fig. 5 shows an example which has a relatively lower volumetric density but higher area percent density. Overall, this may suggest that area and volume density could reflect different aspects of a woman’s breast density and parenchymal pattern, with volumetric density measures reflecting the total amount of dense tissue and area-based density being indicative of the extent of the distribution of the dense tissue within the breast, with an increase in either suggesting increased risk. Further investigation in future prospective studies of the role that different density measures might have in risk assessment may be worthwhile.

In our study, the model fit using only standard risk factors had an AUC of 0.64, which is in range of the 0.58 to 0.67 commonly reported in the literature for models based only on demographic risk factors [11, 21, 41]. Standard area percent density, together with Gail risk factors, had an AUC of 0.68 (95 % CI 0.62-0.74), similar to the 0.64-0.66 range often reported for models incorporating mammographic percent density [17, 21, 40, 42]. Shepherd et al. recently reported that adding percent dense tissue area to a fully adjusted model with fibroglandular tissue volume modestly increased AUC from 0.65 to 0.67 [21]. Although it is not directly comparable, we show that an augmented model, which includes both area and volume density, has an appreciably higher performance (AUC = 0.77; *P* < 0.05), which is further enhanced when also adjusting for BMI (AUC = 0.86; *P* < 0.05). The improved performance in our study may be partially due to the use of digital mammography and the use of fully automated measures of density, allowing more precise estimates, potentially alleviating the bias introduced by intra-reader variability in manual assessment [46] or the digitization of screen-film mammograms or both.

It is worth noting that volumetric breast density methods which estimate breast density from two-dimensional (2D) mammography, such as the one used in this work, do not directly measure the “true” volume of the dense fibroglandular tissue in the breast but rather a surrogate estimate based on imaging physics and validated assumptions of the breast anatomy [47]. Recent work by Wang et al. suggests that volumetric breast density estimates made from 2D mammograms correlate strongly with estimates made from true three-dimensional (3D) images, such as those acquired via MRI [48], although the potential impact this may have on risk assessment has yet to be evaluated. Furthermore, given the increasing interest in the adoption of 3D imaging modalities as a primary screening tool, such as breast tomosynthesis [49], true 3D breast density assessment may soon be feasible for the general screening population. As such, future work should look toward the development and validation of automated breast density assessment tools for 3D screening modalities.

In our study, we found that BMI has a negative correlation to both area and volume percent density (ρ = −0.32 to −0.16) and positive correlations to absolute dense area (ρ = 0.10) and absolute dense volume (ρ = 0.42), which are similar to what has been seen in prior work [50]. Prior studies have also shown that adjusting breast density measures for BMI and other known risk factors can modify the strength of association between density and cancer risk [15, 18, 50]. Specifically, we found that adjusting our univariate models for either Gail risk factors alone (Table 4) or in combination with BMI (Table 5) strengthens the individual associations between most of the fully automated breast density measures and breast cancer. The association between absolute dense area and breast cancer was largely uninfluenced by the inclusion of BMI. Only in the case of absolute dense volume did the inclusion of BMI lead to an attenuated, though still statistically significant, association with breast cancer, likely due to the positive correlation (ρ = 0.42) between the two metrics leading to an overestimation bias. Furthermore, the magnitude of the association observed for BMI in our study was found to be larger than what may be expected on the basis of prior literature [51]. One potential explanation is that our study includes a large fraction of non-Caucasian women, especially African-American (approximately 26 %), which may play a role in our findings as the association between BMI and breast cancer varies by menopausal status, race, and the breast cancer subtype which develops [52]. Larger studies will be needed in order to fully validate the LIBRA density estimates as an independent breast cancer risk factor.

Although there appear to be significant differences between the individual breast density estimates (Table 3), we observed that, as additional risk factors are considered, the predictive capacity of the different models begins to converge (Tables 4 and 5). This is likely due in part to the fact that the different density measures are partly correlated to each other (e.g., the strongest observed correlation being ρ = 0.73 between area percent density and absolute dense area). Furthermore, a common set of risk factors are used to adjust the models reported in Tables 4 and 5. As a result, the various risk factors are expected to potentially capture some similar information about risk which leads to converging AUC scores. However, the fact that the breast density measures reach statistical significance in the logistic regression models adjusted for the standard risk factors and BMI suggests that they also capture some independent information about breast cancer risk. Larger studies are needed in order to fully clarify how breast density should be incorporated into breast cancer risk prediction models in a way that potentially maximizes this independent information.

Pepe et al. proposed five phases for identifying and validating predictive biomarkers [53], in which phases 1 and 2 consist of retrospective analysis to determine the biomarker’s capacity to distinguish between individuals with and without the outcome of interest. In this setting, demonstrating strong associations between a biomarker and the outcome of interest (here, breast cancer), after adjustment for potential confounders, is the strongest evidence that a risk model would ultimately benefit from its inclusion [30]. Therefore, our intention was to perform a preliminary association analysis, with the goal of identifying promising predictors from an array of different novel quantitative measures of breast density which could ultimately have value for breast cancer risk assessment. As such, it is worth noting that our dataset includes a wide spectrum of incident breast cancers, being a representative sample of the general population of women diagnosed with breast cancer.

Limitations of our study include that our analysis is confined to a relatively small sample size and the use of a case–control study design, in which we used contralateral mammograms at the time of diagnosis, rather than prospective follow-up. Of note, certain risk factors known to be associated with breast cancer were not found to be significant in our study, specifically family history, prior biopsy count, and BI-RADS breast density. This is likely because our study was underpowered to reliably detect subtle differences between cases and controls for these measures. Furthermore, it is known that the matching process in case–control studies may introduce biases in assessing biomarker performance [54] because the matching process leads to a skewed distribution of the considered covariates in the study sample relative to the general population [54]. Therefore, the AUCs in our study should be interpreted within the context of the age-matched study design, especially given the individual discriminatory capacity of the BMI risk factor. The AUC comparisons performed in this study must also be considered in the context of our study’s sample size as the sample size required to measure an ROC area difference between two models depends on the correlation of ROC areas for the comparison being made. This may in part explain why the quantitative breast density measures were statistically significant in the logistic regression models, but the increases in AUC of some of the density models were not found to be statistically significant when compared with the baseline risk factor models. As such, our observations will also need to be validated prospectively in larger, independent populations with appropriate adjustment for additional confounders. In addition, although we investigated the association of breast density estimates by using mammograms acquired from a single vendor, additional mammography vendors use different full-field digital mammography technology and will also need to be evaluated with the LIBRA software in order to validate its generalizability. Ultimately, fully automated measures could alleviate the subjectivity in density assessment by visual assessment and provide objective quantitative measures for clinical reporting and guiding personalized screening recommendations.

## Conclusions

We demonstrate that novel fully automated area and volumetric-based breast density measures assessed via digital mammography are associated with breast cancer after adjustment for standard risk factors, such as Gail factors and BMI. These quantitative measures, including volumetric and absolute fibroglandular tissue estimates, could ultimately improve breast cancer risk prediction by providing additional information regarding a woman’s risk for breast cancer, compared with standard risk factors alone. In addition, the ability to fully automate density estimation with digital mammography, particularly through the use of publically available breast density estimation software, could accelerate the translation of density reporting in routine breast cancer screening and surveillance protocols. As such, our observations will be instrumental in guiding the design of larger prospective studies that will more rigorously validate the predictive value of such new fully automated, quantitative, breast density measures in larger populations.

## Additional files

Additional file 1: Table S1. Univariate logistic regression tables for absolute area, absolute volume, VD %, and BI-RADS breast density adjusted for standard risk factors. *BI-RADS* Breast Imaging-Reporting and Data System, *LIBRA* Laboratory for Individualized Breast Radiodensity Assessment, *N/A* not applicable, *VD %* volume percent density. (PDF 207 kb)
Additional file 2: Table S2. Univariate logistic regression tables for absolute area, absolute volume, VD %, and BI-RADS breast density adjusted for both standard risk factors and BMI. *BI-RADS* Breast Imaging-Reporting and Data System, *BMI* body mass index, *LIBRA* Laboratory for Individualized Breast Radiodensity Assessment, *N/A* not applicable, *VD %* volume percent density. (PDF 213 kb)

## Abbreviations

2D: Two-dimensional
3D: Three-dimensional
AUC: Area under the curve
BI-RADS: Breast Imaging-Reporting and Data System
BMI: Body mass index
CI: Confidence interval
LIBRA: Laboratory for Individualized Breast Radiodensity Assessment
MLO: Mediolateral oblique
OR: Odds ratio
PD %: Area percent density
ROC: Receiver operating characteristic
VD %: Volume percent density
