# Supplementary material for: Preliminary evaluation of the publicly available Laboratory for Breast Radiodensity Assessment (LIBRA) software tool: comparison of fully automated area and volumetric density measures in a case–control study with digital mammography
Source: Breast Cancer Res. 2015 Aug 25;17:117. doi: 10.1186/s13058-015-0626-8 (PMC4549121; doi:10.1186/s13058-015-0626-8)
Supplement: Additional file 1: Table S1. — Univariate logistic regression tables for absolute area, absolute volume, VD %, and BI-RADS breast density adjusted for standard risk factors. BI-RADS Breast Imaging-Reporting and Data System, LIBRA Laboratory for Individualized Breast Radiodensity Assessment, N/A not applicable, VD % volume percent density. (PDF 207 kb) [file 13058_2015_626_MOESM1_ESM.pdf]

**Additional file 1: Table S1**. Logistic regression models and discriminatory capacity of the risk factor adjusted breast density estimates not already shown in Table 6. Odds ratios, area under the curve (AUC) of the receiver operating characteristic (ROC) and 95% confidence intervals for the models are provided. Dashes indicate features not included in each model.

| Risk Factor | Gail Risk Factors Plus Absolute Dense Area | | | Gail Risk Factors Plus Absolute Dense Volume | | | Gail Risk Factors Plus Volume Percent Density | | | Gail Risk Factors Plus BI-RADS Breast Density | | |
|---|---|---|---|---|---|---|---|---|---|---|---|---|
| | Odds Ratio | p-value | AUC | Odds Ratio | p-value | AUC | Odds Ratio | p-value | AUC | Odds Ratio | p-value | AUC |
| **Demographics** | | | | | | | | | | | | |
| **Age** | 1.03 (0.89-1.18) | 0.71 | | 1.05 (0.91-1.21) | 0.49 | | 1.03 (0.90-1.19) | 0.63 | | 0.96 (0.84-1.10) | 0.60 | |
| **Age at Menarche** | 0.59 (0.37-0.94) | 0.03 | | 0.65 (0.41-1.03) | 0.07 | | 0.56 (0.35-0.88) | 0.01 | | 0.56 (0.35-0.88 | 0.01 | |
| **Number of Benign Biopsies** | 1.00 (0.68-1.48) | 0.99 | | 0.99 (0.67-1.46) | 0.96 | | 0.99 (0.68-1.43) | 0.94 | | 1.08 (0.75-1.55) | 0.69 | |
| **1st Degree Family History** | 0.76 (0.44-1.31) | 0.32 | | 0.76 (0.45-1.31) | 0.33 | | 0.84 (0.50-1.42) | 0.52 | | 0.92 (0.55-1.53) | 0.74 | |
| **Parity** | | | | | | | | | | | | |
| Nulliparous | 1 (N/A) | - | | 1 (N/A) | - | | 1 (N/A) | - | | 1 (N/A) | - | |
| Prior to age 30y | 2.89 (1.48-5.68) | 0.002 | | 3.21 (1.63-6.32) | 0.001 | | 2.69 (1.41-5.14) | 0.003 | | 0.24 (1.19-4.21) | 0.01 | |
| Age 30y or older | 2.29 (1.07-4.88) | 0.03 | | 2.41 (1.13-5.13) | 0.02 | | 2.24 (1.09-4.61) | 0.03 | | 2.01 (0.99-4.08) | 0.05 | |
| **Race** | | | 0.75 (0.70-0.81) | | | 0.74 (0.68-0.79) | | | 0.68 (0.62-0.74) | | | 0.64 (0.58-0.71) |
| Caucasian | 1 (N/A) | - | | 1 (N/A) | - | | 1 (N/A) | - | | 1 (N/A) | - | |
| African-American | 0.47 (0.25-0.88) | 0.02 | | 0.36 (0.19-0.69) | 0.002 | | 0.52 (0.29-0.94) | 0.03 | | 0.54 (0.30-0.97) | 0.04 | |
| Asian | 0.65 (0.12-3.52) | 0.62 | | 0.42 (0.08-2.13) | 0.30 | | 0.39 (0.08-1.91) | 0.24 | | 0.45 (0.09-2.20) | 0.37 | |
| Other | 1.07 (0.23-4.93) | 0.93 | | 0.90 (0.21-3.84) | 0.89 | | 0.87 (0.21-3.71) | 0.85 | | 0.81 (0.20-3.34) | 0.77 | |
| **Continuous Density Estimates** | | | | | | | | | | | | |
| ***LIBRA:*** Absolute Dense Area | 2.45 (1.86-3.24) | <0.001 | | - | - | | - | - | | - | - | |
| ***Quantra:*** Absolute Dense Volume | - | - | | 2.40 (1.80-3.21) | <0.001 | | - | - | | - | - | |
| ***Quantra:*** Volume Percent Density | - | - | | - | - | | 1.69 (1.27-2.22) | <0.001 | | - | - | |
| **BI-RADS Breast Density** | - | - | | - | - | | - | - | | 1.07 (0.74-1.56) | 0.71 | |
