# Supplementary material for: Preliminary evaluation of the publicly available Laboratory for Breast Radiodensity Assessment (LIBRA) software tool: comparison of fully automated area and volumetric density measures in a case–control study with digital mammography
Source: Breast Cancer Res. 2015 Aug 25;17:117. doi: 10.1186/s13058-015-0626-8 (PMC4549121; doi:10.1186/s13058-015-0626-8)
Supplement: Additional file 2: Table S2. — Univariate logistic regression tables for absolute area, absolute volume, VD %, and BI-RADS breast density adjusted for both standard risk factors and BMI. BI-RADS Breast Imaging-Reporting and Data System, BMI body mass index, LIBRA Laboratory for Individualized Breast Radiodensity Assessment, N/A not applicable, VD % volume percent density. (PDF 213 kb) [file 13058_2015_626_MOESM2_ESM.pdf]

Additional file 2: Table S2. Logistic regression models and discriminatory capacity of the risk factor and BMI adjusted breast density estimates not already shown in Table 7. Odds ratios, area under the curve (AUC) of the receiver operating characteristic (ROC) and 95% confidence intervals for the models are provided. Dashes indicate features not included in each model.

Risk Factor	Gail Risk Factors and BMI Plus Absolute Dense Area			Gail Risk Factors and BMI Plus Absolute Dense Volume			Gail Risk Factors and BMI Plus Volume Percent Density			Gail Risk Factors and BMI Plus BI-RADS Breast Density		
	Odds Ratio	p-value	AUC	Odds Ratio	p-value	AUC	Odds Ratio	p-value	AUC	Odds Ratio	p-value	AUC
**Demographics**												
**Age**	1.06 (0.90-1.25)	0.48		1.05 (0.89-1.23)	0.57		1.09 (0.92-1.29)	0.31		0.98 (0.83-1.14)	0.77	
**Age at Menarche**	0.80 (0.48-1.34)	0.40		0.78 (0.47-1.29)	0.33		0.73 (0.44-1.21)	0.22		0.72 (0.44-1.20)	0.21	
**Number of Benign Biopsies**	1.13 (0.74-1.73)	0.58		1.10 (0.73-1.68)	0.64		1.06 (0.70-1.62)	0.78		1.19 (0.79-1.78)	0.40	
**1st Degree Family History**	0.77 (0.42-1.41)	0.40		0.80 (0.45-1.42)	0.44		0.78 (0.43-1.39)	0.55		0.89 (0.51-1.57)	0.70	
**Parity**												
Nulliparous	1 (N/A)	-		1 (N/A)	-		1 (N/A)	-		1 (N/A)	-	
Prior to age 30y	2.64 (1.25-5.56)	0.01		2.80 (1.34-5.85)	0.006		2.68 (1.28-5.63)	0.009		2.09 (1.04-4.20)	0.04	
Age 30y or older	2.13 (0.92-4.92)	0.08		2.37 (1.04-5.38)	0.04		2.34 (1.03-5.32)	0.04		2.00 (0.91-4.40)	0.08	
**BMI**	3.62 (2.36-5.56)	<0.001	0.85 (0.81-0.90)	2.89 (1.92-4.33)	<0.001	0.82 (0.77-0.86)	4.14 (2.65-6.45)	<0.001	0.83 (0.78-0.87)	3.43 (0.68-2.03)	<0.001	0.80 (0.75-0.85)
**Race**												
Caucasian	1 (N/A)	-		1 (N/A)	-		1 (N/A)	-		1 (N/A)	-	
African-American	0.25 (0.11-0.56)	0.001		0.24 (0.11-0.51)	<0.001		0.26 (0.12-0.56)	0.001		0.30 (0.14-0.63)	0.001	
Asian	0.85 (0.13-5.36)	0.86		0.55 (0.10-3.16)	0.51		0.51 (0.09-3.02)	0.46		0.58 (0.10-3.39)	0.55	
Other	0.96 (0.18-0.05)	0.97		0.83 (0.17-4.08)	0.82		0.83 (0.15-4.74)	0.84		0.74 (0.45-3.75)	0.72	
**Continuous Density Estimates**												
LIBRA: Absolute Dense Area	2.57 (1.86-3.56)	<0.001		-	-		-	-		-	-	
Quantra: Absolute Dense Volume	-	-		1.96 (1.44-2.67)	<0.001		-	-		-	-	
Quantra: Volume Percent Density	-	-		-	-		2.24 (1.56-3.21)	<0.001		-	-	
**BI-RADS Breast Density**	-	-		-	-		-	-		1.06 (0.69-1.63)	0.78	
